# Regulating the Regulators: Immune System Regulators Are Highly Susceptible to HIV Infection

**DOI:** 10.1371/journal.pbio.0020209

**Published:** 2004-07-13

Nearly a dozen varieties of interdependent cells work in harmony to protect the body from infectious pathogens. Dendritic cells and B-cells carry remnants of pathogens to nearby helper T-cells (also known as CD4 cells because they express the CD4 protein on their surfaces), which coordinate a threat response by signaling killer T-cells to destroy the intruder. Yet another class of cells cleans up the debris such an encounter inevitably creates. Each of these cell types is further classified based on the combinations of surface proteins they express. Such proteins may simply identify the cell type or may help define its function.[Fig pbio-0020209-g001]

**Figure pbio-0020209-g001:**
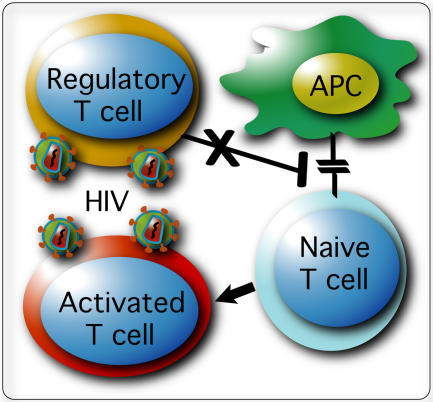
Inhibition of Treg function by HIV leads to T-cell hyperactivation

An immune response can activate millions of cells; once these cells perform their respective jobs, it's crucial that they retire from active duty. That's where regulatory T-cells, or Treg cells, come in: an immune system that fails to retreat from an immune response can be just as dangerous as one that fails to organize.

Research has recently focused on a subset of Treg cells that express CD4 and CD25 proteins (so-called CD4^+^CD25^+^ T-cells) and that suppress T-cell activation in mice and humans. Treg cells appear to be key immune system regulators, as their absence results in autoimmune and allergic diseases. Now a team led by Derya Unutmaz at Vanderbilt University report that human Treg cells are highly vulnerable to HIV infection.

Treg cells isolated from healthy volunteers were not only highly susceptible to HIV infection but were also killed by the virus. Because Treg cells account for only about 1% of human T-cells and are difficult to grow in a test tube, Unutmaz and colleagues developed a way to manufacture sufficient quantities for study by introducing a transcription factor called *FoxP3* into conventional “naïve” T-cells (cells not yet primed to recognize a specific target). *FOXP3* is required for the development of Treg cells in mice. Though it's not clear what role the human form of the gene plays in human Treg cell development, *FOXP3* mutations cause an autoimmune disease associated with hyperactive T-cells. Here the authors demonstrate that *FoxP3* transforms T-cells into Treg cells. The *FoxP3*-engineered T-cells behaved just like naturally occurring Treg cells: when exposed to a population of resting naïve CD4 T-cells, the engineered cells suppressed their activation. Overexpression of *FoxP3* also made activated T-cells more susceptible to infection.

Since Treg cells are so susceptible to HIV, the researchers reasoned that these cells might be compromised in HIV-infected patients and that loss of Treg cells could lead to T-cell hyperactivation. Their logic was borne out by the finding that a portion of HIV patients with low CD4 counts and high levels of activated T-cells also had greatly depleted numbers of *FoxP3*-expressing CD4^+^CD25^+^ T-cells—a marker for Treg cells. The finding that HIV targets cells that normally suppress immune function is significant, given that HIV infection is characterized by chronic T-cell hyperactivation. Disruption of Treg cells, Unutmaz and colleagues conclude, could in turn disrupt the delicate balance of immune system function, setting the stage for hyperactivation—and a chronically hyperactive immune response could eventually exhaust the immune system. With a method to generate large numbers of Treg cells for study, Unutmaz and colleagues have paved the way for identifying mechanisms that mediate Treg cells' suppressive function and provided another resource for determining how HIV tips the scales toward disease progression.

